# Disease named entity recognition by combining conditional random fields and bidirectional recurrent neural networks

**DOI:** 10.1093/database/baw140

**Published:** 2016-10-24

**Authors:** Qikang Wei, Tao Chen, Ruifeng Xu, Yulan He, Lin Gui

**Affiliations:** 1 Shenzhen Engineering Laboratory of Performance Robots at Digital Stage, Harbin Institute of Technology Shenzhen Graduate School, Shenzhen, China and; 2 School of Engineering and Applied Science, Aston University, Birmingham, UK

## Abstract

The recognition of disease and chemical named entities in scientific articles is a very important subtask in information extraction in the biomedical domain. Due to the diversity and complexity of disease names, the recognition of named entities of diseases is rather tougher than those of chemical names. Although there are some remarkable chemical named entity recognition systems available online such as ChemSpot and tmChem, the publicly available recognition systems of disease named entities are rare. This article presents a system for disease named entity recognition (DNER) and normalization. First, two separate DNER models are developed. One is based on conditional random fields model with a rule-based post-processing module. The other one is based on the bidirectional recurrent neural networks. Then the named entities recognized by each of the DNER model are fed into a support vector machine classifier for combining results. Finally, each recognized disease named entity is normalized to a medical subject heading disease name by using a vector space model based method. Experimental results show that using 1000 PubMed abstracts for training, our proposed system achieves an F1-measure of 0.8428 at the mention level and 0.7804 at the concept level, respectively, on the testing data of the chemical-disease relation task in BioCreative V.

**Database URL:** http://219.223.252.210:8080/SS/cdr.html

## Introduction

With the rapid increase of biomedical literature, it is crucial to facilitate automatic recognition of chemical and disease named entities from text since knowledge discovered is important to a number of biomedical related applications such as drug discovery, safety surveillance and drug side effect detection. Manual recognition of named entities, though gives high extraction accuracy, is labor intensive. Therefore, there is an urgent need to develop an automatic annotation system based on natural language processing (NLP) techniques.

Many systems for disease and chemical entity recognition from text were developed (1, 2). Some of these relied on biomedical dictionaries. For example, the Jochem dictionary (3) employed a lexical approach to recognize the diverse representation of chemical information in literatures; a hybrid system called ChemSpot (4) also used the lexical-based approach to locate chemical named entities. Systems based on machine learning and large training corpora were also developed. Klinger *et al.* (5) employed conditional random fields (CRFs) to find the International Union of Pure and Applied Chemistry (IUPAC) and IUPAC-like chemical names; Leaman and Gonzalez (6) presented BANNER, which is an open-source biomedical named entity recognition system implemented using CRFs; Leaman *et al.* (7) developed a high performance chemical named entity recognizer created by combining two independent machine learning models in an ensemble. Currently, there are some remarkable chemical named entity recognition systems available online. However, the publicly available recognition systems of disease named entities are rare mainly due to the diversity and complexity of disease named entities in naming conventions and the lack of appropriate training corpora. Furthermore, the disease name entities often have many naming variations. This further complicates the recognition and normalization of disease entities.

Variants of CRF-based approaches have been successfully applied to named entity recognition (NER). The main drawback of these models is that many of them focused on designing hand-crafted features which is labor intensive. Also, features defined are often domain- and data-specific. Recently, deep neural network models, which can automatically extract features from free-text data, have attracted significant attention. Chiu and Nichols (8) presented a novel neural network architecture that automatically detected word- and character-level features using a hybrid bidirectional long short term memory (LSTM) network and a convolutional neural network, eliminating the need for feature engineering. Santos and Guimaraes (9) proposed a CharWNN deep neural network, which used word- and character-level representations (embeddings) to perform sequential classification. Lample *et al.* (10) introduced two new neural architectures: one based on bidirectional LSTM and CRFs and the other that constructed and labeled segments using a transition-based approach inspired by shift-reduce parsers. Irsoy and Cardie (11) applied deep stacked bidirectional recurrent neural network (Bi-RNN) to the task of opinion expression extraction formulated as a token-level sequence-labeling task.

The chemical-disease relation (CDR) task (1) in BioCreative V aims to encourage the further development of techniques for recognizing chemical and disease entities and detecting the CDRs. Disease named entity recognition (DNER) and normalization is an intermediate step before the CDR extraction. In this subtask, each participant system is required to return recognized mentions and their exact locations. Furthermore, the normalized disease concept identifiers assigned by medical subject headings (MeSHs) for the given PubMed (12) abstract are also required.

In this article, we introduce a machine learning based DNER and normalization approach. First, two separate DNER models are developed. One model is based on CRFs with a rule-based post-processing module. The other one is based on Bi-RNN. The recognized named entities by each model are fed into a support vector machine (SVM) classifier for combining results. Finally, dictionary matching and vector space model (VSM) based normalization method are used to align the recognized mention-level disease named entities with concepts in MeSH.

In the evaluation, using the 1000 PubMed abstracts as the training dataset, our system achieves an F1-measure of 0.8428 at the mention level and 0.7804 at the normalized concept level, respectively, on the testing data of the CDR task in BioCreative V. Furthermore, the achieved performance is higher than any of the CRFs- or Bi-RNN-based DNER models, which shows the benefit of using SVM for combining results. We have made a web service of our system available at http://219.223.252.210:8080/SS/cdr.html. 

## Materials and methods

### Subtask 1: DNER

For the DNER subtask of the CDR task in BioCreative V, each participant system is required to recognize the disease named entities from given raw PubMed abstracts automatically in limited time.

### Dataset

To assist the system development and assessment, the organizer has manually annotated all chemicals, diseases and their interactions in 1500 PubMed abstracts. For the CDR task, training (500 abstracts) and development (500 abstracts) datasets have been released by the task organizers (13). Besides, 500 raw abstracts are used as the test set for system evaluation. Each dataset contains PubMed abstracts, named entity annotations, start-end position and their aligned concept ID from the MeSH database. For the DNER subtask, each participant system is required to recognize the disease named entities. Table 1 gives the statistics of the released data of the DNER subtask.

**Table 1.** Statistics of the released data of the DNER subtask in the DR task

Dataset	Abstract	Disease named entity	
		Mentions	Concept IDs
Training set	500	4182	1965
Development set	500	4244	1865
Test set	500	4424	1988

### Architecture

**Figure 1.**
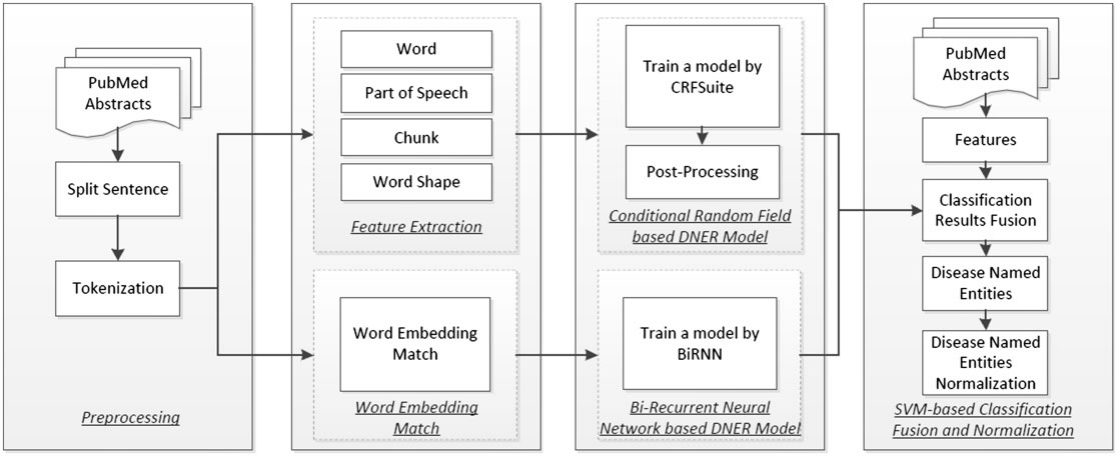
System architecture.

Our system consists of four basic components, as shown in Figure 1: (i) preprocessing of the PubMed corpus: we use a sentence boundary detection tool called ‘Splitta’ (https://code.google.com/archive/p/splitta/) to split each PubMed abstract into sentences which are subsequently tagged using the GENIA Tagger (14); (ii) feature extraction: this component extracts the features from the preprocessed PubMed text, including words, part-of-speech tags, chunking information, word shape features such as dictionary and morphological features and word embeddings. The first four types of features will be used in the CRFs model, while the word embeddings will be used in the Bi-RNN model; (iii) the base DNER models: two DNER models, based on CRFs with rule-based post-processing and Bi-RNN, respectively, are trained and applied to recognize disease named entity from PubMed text and (iv) recognition outputs combination and normalization: the outputs from the two base DNER models and some additional features are fed into a SVM-based classifier to generate optimized outputs. Finally, the named entity normalization component uses dictionary matching and a VSM-based method to align the recognized mention-level disease named entities with concepts in MeSH.

### DNER using CRFs

**Figure 2.**
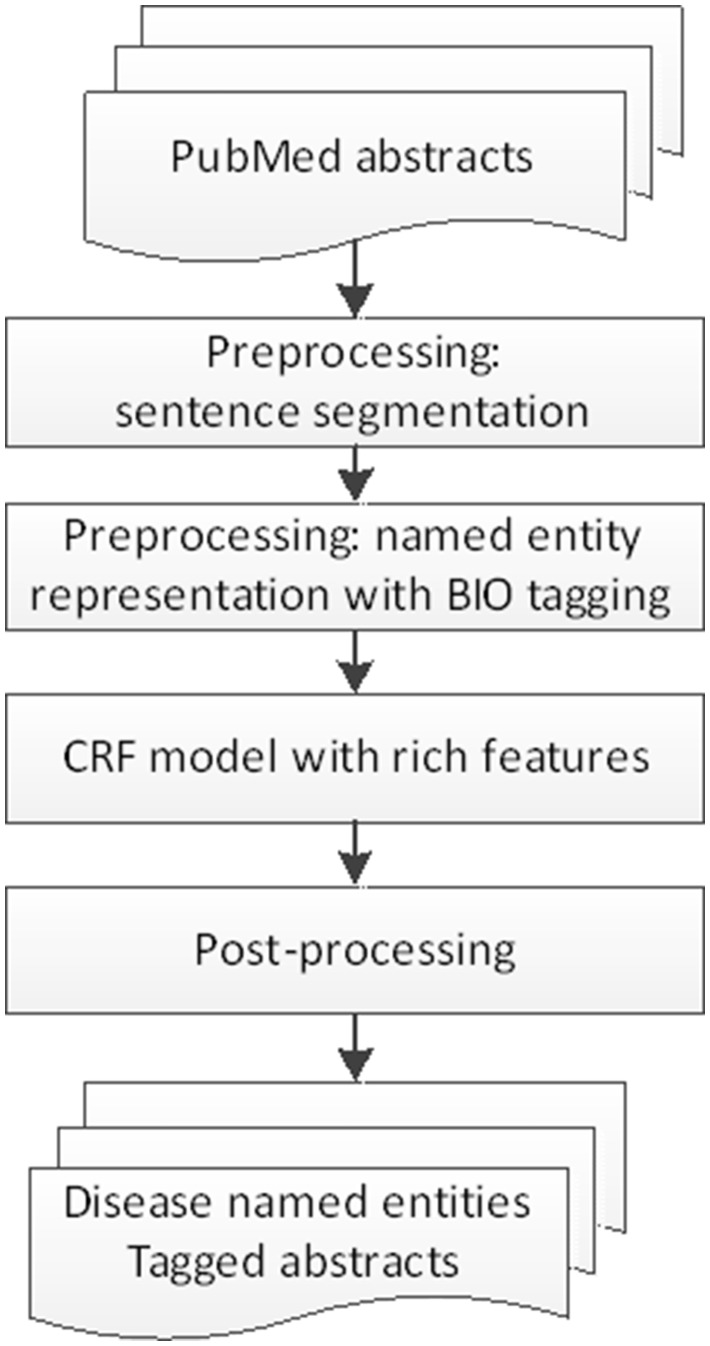
The flow chart of CRF-based DNER model.

The first DNER model is based on CRFs. The flow chart of the CRF model is described in Figure 2.

In this model, first the chemical named entities and the chemical-induced disease (CID) relations are removed. The PubMed abstracts are then split into sentences by using ‘Splitta’, from which features are extracted for training the CRF-based DNER model.

The feature set is listed below:

- Word: The word itself.
- POS: The part-of-speech tag of each word which is generated by the GENIA tagger.
- Chunk: The chunking information for each word which is generated by the GENIA tagger.
- Word Shape: Here, we represent each word by its shape information. If a character is in upper case, we represent it as ‘*U*’, lower case as ‘*L*’ and digit as ‘*D*’, etc. For example, a disease name ‘delirium’ will be represented as ‘LLLLLLLL’ (15).
- Type: Classify each word to different types such as ‘All Digit’, ‘All Symbol’, ‘All Upper and Digit’, ‘All Letter’, etc.
- Prefix and Suffix: The 1∼4 prefix and suffix of each word are used as feature.
- Dictionary Look-up feature: We extract all the disease names in the Unified Medical Language System (UMLS) Meta thesaurus MeSH database as a disease dictionary and use it in two different ways: 1) we split all the disease named entities in the corpus into single words. If any of the words can be found in the disease dictionary, we mark a ‘Y’ in its feature representation and ‘N’ otherwise; 2) we traverse the corpus and perform dictionary look-up for each word. Words are annotated with ‘Begin’, ‘In’ and ‘Out’ (BIO) tags based on longest common subsequence matching.

In this model, the *(−n, n)* words are selected as the context window of the observing word, where *n* varies from 1 to 3 based on the type of the features.

The CRFSuite (16), which is very fast among the various implementations of CRFs, is adopted to develop this CRF-based DNER model.

**Figure 3.**
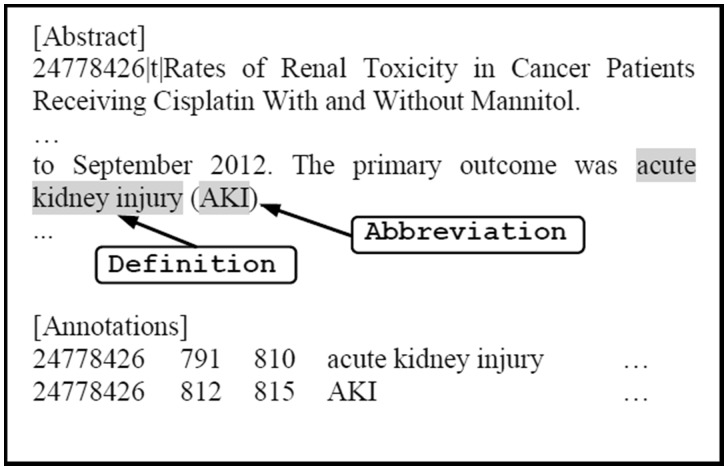
A Sample output of our abbreviation resolution.

To further improve the performance of the CRF-based DNER model, post-processing techniques are applied. We tag all instances of a certain entity as a disease name mention if this entity is tagged by the CRF-based DNER more than twice within an abstract (15). For example, if ‘psychosis’ is tagged as a disease named entity twice in one abstract, it indicates this word is very likely to be a disease named entity. Hence, all of the occurrences of ‘psychosis’ are labeled as disease named entity. We also perform abbreviation resolution by using the script (Available at: http://www.cnts.ua.ac.be/∼vincent/scripts/abbreviations.py) developed based on the algorithm proposed by Schwartz and Hearst (17). It detects links between an abbreviation and its definition, if they are in the form of ‘<definition> (<abbreviation>)’. The process here consists of two steps: (i) traversing the abstract sentences and locating the word sequences in the form of ‘<definition> (<abbreviation>)’ and (ii) looking up the definition in a custom UMLS disease dictionary. If the definition can be found in the dictionary, we tag its abbreviation as a disease entity. As illustrated in Figure 3, our CRF model correctly tagged ‘acute kidney injury’ as a disease name but failed to recognize that ‘AKI’ is also a disease name. Such an error would be corrected by abbreviation resolution since ‘acute kidney injury (AKI)’ follows the form of ‘<definition> (<abbreviation>)’ and ‘acute kidney injury’ can be found in the disease dictionary. Hence, ‘AKI’ would be tagged as a disease entity as well. These post-processing techniques are expected to improve the recall of the system.

### DNER using Bi-RNN

RNN is a class of artificial neural network where connections between units form a directed cycle (https://en.wikipedia.org/wiki/Recurrent_neural_network#Bi-directional_RNN). It takes arbitrary sequences as input, uses its internal memory network to exhibit dynamic temporal behavior. It was first used in handwriting recognition and speech recognition. With the rapid development of deep neural networks and parallel computing, there are increasing interests in using RNNs for NLP tasks including NER.

Bi-RNN, invented by Schuster and Paliwal (18), uses a finite sequence to predict or label each element of the sequence based on both the past and the future context of the element. This is done by adding the outputs of two RNNs while one processing the sequence from left to right, the other from right to left. The combined outputs are the predictions of the teacher-given target signals.

**Figure 4.**
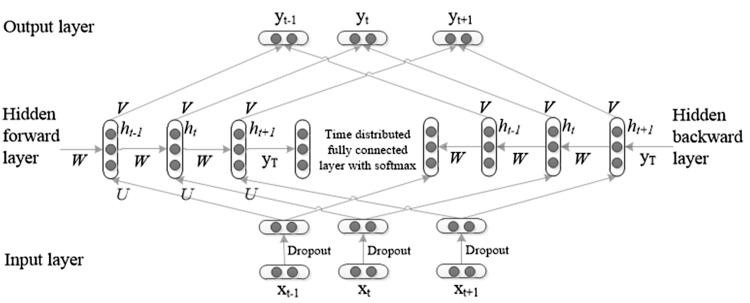
An illustration of Bi-RNN.

As illustrated in Figure 4, a Bi-RNN is a neural network that takes sequential data of variable length *x* = (*x*_1_, …, *x_T_*) as input, consists of a hidden forward layer and a hidden backward layer with hidden unit function *h*, and an optional output *y*. At each time step *t*, the hidden state *h_t_* of the hidden forward layer is computed based on the previous hidden state *h_t_*_−__1_ and the input at the current step *x_t_*:

(1) $$h_{t}=f(Ux_{t}+Wh_{t-1}+b)$$

where *U, W* and *b* are parameter matrices of the network. *T* is the last time step *f(·)*is a non-linear activation function, which can be as simple as an element-wise logistic sigmoid function or as complex as a LSTM unit (19) or Gated Rucurrent Unit (GRU) (20).

At each time step *t*, the hidden state *h_t_* of the hidden backward layer is computed based on the future hidden state *h_t + _*_1_ and the input at the current step *x_t_*:

(2) $$h_{t}=f(Ux_{t}+Wh_{t+1}+b)$$

Afterwards, we add a time distributed fully connected layer with softmax to convert real values to conditional probabilities, which is calculated as follows:

(3) $$P_{i}=\frac{\;\text{exp}\;(x_{i})}{\sum_{i\prime =1}^{C}\;\text{exp}\;(x_{i\prime })}$$

where *C* is the class number.

The output at step *t* is computed as follows:

(4) $$y_{t}=softmax\left(Vh_{t}\right)$$

where *V* is another weight parameter of the network.

Note that the forward and backward parts of the network are independent of each other until the output layer when they are combined. This means that during training, after back-propagating the error terms from the output layer to the forward and backward hidden layers, the two parts can be thought of as separate and each trained with the classical back-propagation through time (11).

Before training a Bi-RNN for DNER, continuous skip-gram model proposed by Mikolov *et al.* (21) is used in to train initial word embeddings from a large PubMed (http://www.ncbi.nlm.nih.gov/pubmed) corpus. It is a data-driven model which takes unstructured text data as input, outputs dense and real-valued vectors in a low-dimensional space (word embeddings) to represent syntactic and semantic word relationships of the words in the input text. The word2vec (https://code.google.com/p/word2vec/) toolkit is use to build the continuous skip-gram model. Other pre-trained word embeddings, e.g. the public available word embeddings trained from the Google News dataset (∼100 billion words), can also be used as the initial word vector inputs into Bi-RNN.

For training a Bi-RNN for DNER, we use the tools provided by (11) (https://github.com/oir/deep-recurrent). The input sequence *x_t_* is set to the *t*-th word embedding in an input sentence; *U*, *W*, *V* and *h*_0_ can be initialized to a random vector of small values, *h_t_*_ + 1_ can be initialized to a copy of *h_t_* recursively. A back-propagation algorithm with Adam stochastic optimization method is used to train the network through time. After each training epoch, the network is tested on validation data. The log-likelihood of validation data is computed for convergence detection. To prevent the models from depending on one dataset too strongly, dropout regularization (dropout = 0.25), introduced by Hinton *et al.* (22), is used on the input sequences.

### Combining the outputs of two models by using SVMs

Bagging outputs from multiple classifiers is commonly used to improve the performance of individual classifiers. In this article, we explore the use of SVMs for combining outputs from the CRF-based DNER and the Bi-RNN-based DNER model. LIBSVM tools provided by Chang and Lin (23) is used in our system.

The classification error rate of each base DNER model on the training data is estimated to generate the weighted confidence scores of the DNER outputs. For a candidate DNER model, its classification results with corresponding confidence scores are used as the input to a SVM classifier to generate the final output. We take each word as an instance for classification. The feature set is listed below:

- Labels: The prediction labels from each base DNER model.
- Confidences: The confidence scores of each base DNER model outputs.
- Error rate: The classification error rate of each base DNER model.
- POS tags: The part-of-speech tag of each word, which is generated by the GENIA tagger.
- Dictionary Look-up feature: Same as the dictionary feature used in the CRF model.
- Word embedding feature: Word embedding generated in the penultimate layer of the Bi-RNN model.

**Figure 5.**
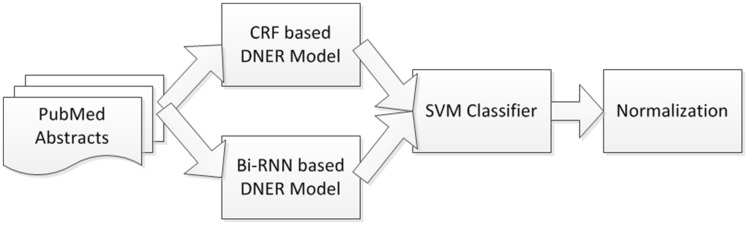
Architecture of the SVM-based model.

Figure 5 shows the architecture of the SVM-based component for combining results from two DNER models.

### Disease named entity normalization module

In the normalization step, we develop a VSM-based method to find the correct MeSH ID for a given entity. The disease entity is used as a query and each MeSH term including its synonyms is considered as one document. To collect one concept’s synonyms as many as possible, for each MeSH ID, we first find its Concept Unique Identifier (CUI) ID in UMLS, treat all the English terms that have the same CUI as the MeSH ID’s document term. All of the words in each document and query are stemmed and changed to lower case. We then calculate the term frequency-inverse document frequency (TF-IDF) of these entities and documents, respectively, and use cosine similarity measure to rank the candidate documents. Finally, the top ranked MeSH ID is selected as the correct ID of the entity. If there is more than one MeSH ID with the highest similarity score, we randomly select one of them as the correct ID; and if the highest score is 0, we mark ‘−1’ as required. 

## Results

In this evaluation, performance is reported as precision, recall and F-measure.

Table 2 gives the performance achieved by the CRF-based model on the 500-abstract test set in the DNER subtask.

**Table 2.** The performance of CRF-based DNER model (The best result is highlighted in bold face)

Different features of CRF model	DNER		
	Precision (%)	Recall (%)	F-measure (%)
Experiment A	82.52	72.22	77.03
Experiment B	85.01	80.65	82.77
Experiment C	85.11	80.76	**82.88**

The difference between Experiments A–C is the use of the dictionary look-up features. In Experiment A, the dictionary look-up features come from the disease terms extracted from the MeSH while each single word is tagged as Y or N. In Experiment B, the dictionary features come from the disease terms extracted from the same database, each word is tagged by ‘BIO’ based on a longest common subsequence matching. In Experiment C, we use both the dictionary features in Experiments A and B. The experimental results show that the third choice of the dictionary look-up features performs better.

Table 3 gives the performance achieved by the Bi-RNN-based model on the 500-abstract test set in the DNER subtask.

**Table 3.** The performance of Bi-RNN-based model (The best result is highlighted in bold face)

RNN model	DNER		
	Precision (%)	Recall (%)	F-measure (%)
RNN with Vectors A	70.76	67.40	69.04
Bi-RNN with Vectors A	74.96	75.74	75.35
Bi-RNN with Vectors B	77.47	79.09	**78.27**

**Table 4.** The performance of output fusion by SVM (The best result is highlighted in bold face)

Model	DNER		
	Precision (%)	Recall (%)	F-measure (%)
CRF model Experiment C	85.11	80.76	82.88
Bi-RNN using Vectors B	77.47	79.09	78.27
Output fusion by SVM	85.28	83.30	**84.28**
Baseline^a^	40.88	59.95	48.61

a The baseline provided by the organizer is based on dictionary look up.

The ‘RNN model with Vectors A’ and ‘Bi-RNN model with Vectors A’ utilize word vectors pre-trained on part of the Google News dataset (∼100 billion words). There are altogether 3 million vectors of words and phrases, each of which contains 300 dimensions. The phrases are obtained by using a simple data-driven approach described in (21). The hidden layers of both RNN and Bi-RNN are set to 500, and the dropout is set to 0.25.

The ‘Bi-RNN model with Vectors B’ utilizes vectors pre-trained on a large PubMed corpus. The PubMed comprises >25 million citations for biomedical literature from MEDLINE, life science journals and online books. These word vectors also have 300 dimensions.

The statistical analysis shows that the words in the vectors trained by Google News cover around 50% of the vocabulary in the CDR corpus while the vectors trained by PubMed articles cover 76% of the vocabulary of the CDR corpus. That is the main reason that ‘Bi-RNN with Vectors B’ performs better. Furthermore, it is also observed from Table 3 that, Bi-RNN improves over RNN significantly on the DNER task.

We choose the ‘Experiment C’ setup in CRFs and ‘Bi-RNN using Vectors B’ as the base DNER models. Their outputs are fed into a SVM classifier for combining results. It is observed from Table 4 that the combined outputs improve over the CRF-based model by 1.40% and the Bi-RNN model by 6.01%, respectively. Finally, our approach achieves 84.28% in F-measure at the mention level which is much higher than the 48.51% in F-measure by the baseline.

We compare the different features used in our system. Word embedding features lead to a significant improvement for all the three model of our system on all the three metrics. Dictionary look-up features are also informative for performance improvement. The prediction labels and confidences features from CRF and Bi-RNN contributed a higher performance enhancement (84.28 vs. 82.88% for CRF, 84.28 vs. 78.27% for Bi-RNN).

The concept-level DNER performance is also evaluated. The obtained results are listed in Table 5. It is observed that our final system achieves F1 performance of 78.04% at the concept level which is higher than the baseline based on dictionary look up by 25.74%.

**Table 5.** The concept-level DNER performance

Model	Disease name entity normalization		
	Precision (%)	Recall (%)	F-measure (%)
Our approach	76.57	79.57	78.04
Baseline	42.71	67.46	52.30

We have examined the efficiency of each model and the overall system using a computer with 32 GB RAM and a 3.6 GHz 8-core processor. It took <15 min to train CRF model for DNER, ∼8 h to train Bi-RNN model for DNER and ∼15 min to train SVM model. For testing on the overall system, the average processing time for one sentence was <20 s, in which most of time was used to load the trained model.

## Error analysis and discussion

In the disease NER task, we develop a CRF and a Bi-RNN-based NER system, and then combines their outputs by SVM. The errors in boundary detection led to a main performance loss. Some of the boundary errors come from a redundant modifier, e.g. our system recognized ‘intestinal bleeding’ instead of ‘bleeding’, ‘cardiac chest pain’ instead of ‘chest pain’; some come from a missed modifier, e.g. we find ‘depression’ instead of ‘major depression’, ‘vasculitis’ instead of ‘ANCA positive vasculitis’. Beyond that, the limited ability in handling the abbreviations also leads to some errors, especially when the fully spelled mention is not considered as an entity but the abbreviation is. For example, ‘OIH’ is the short form of ‘Opioid-induced hyperalgesia’. In our abbreviation resolution method, if the original phrase, ‘Opioid-induced hyperalgesia’, is not detected as a disease named entity, then the abbreviation ‘OIH’ in the parentheses won’t be detected either. Similarly, the abbreviation of disease named entity ‘HITT’ is also missed.

In the normalization task, the difficulty in dealing with a sequence IDs such as ‘D014786|D034381 vision loss|hearing loss’ for ‘vision and hearing loss’ led to some errors. Besides, if the system fails to find the correct ID, that would also cause a performance drop.

It is worth mentioning that since disambiguation is not critical for DNER, it is not performed in our current experiments.

## Conclusions

This article presents a system for recognizing and normalizing the disease named entities. First, a CRF-based DNER model is developed for recognizing disease named entities. It employs rich features including orthographic, morphological and domain knowledge from UMLS. Rule-based post-processing techniques are then employed to identify the missed entities. Second, a Bi-RNN-based model with domain-specific word embeddings is developed. Next, a SVM classifier is applied to combine the results from two individual DNER models to further improve the performance of DNER. Finally, the recognized disease named entity is normalized to a MeSH disease name by a VSM-based method. The evaluations on the CDR dataset in BioCreative V shows that our approach achieves 84.28% in F-measure at the mention level which is much higher than the baseline and the results of two individual models. Nevertheless, for concept-level DNER, our approach only gives an F-measure of 78.04%. Further improvements include using other domain knowledge and optimized features for CRF training, employing better strategies for outputs combination and investigating better methods for normalization.

## Funding

This work is supported by the National Natural Science Foundation of China (No. 61370165, No. 61632011); National 863 Program of China (2015AA015405); Shenzhen Fundamental Research Funding (JCYJ20150625142543470); Shenzhen Peacock Plan Research Grant (KQCX20140521144507925) and Guangdong Provincial Engineering Technology Research Center for Data Science (2016KF09). National Natural Science Foundation of China pay for the open access publication.

*Conflict of interest*. None declared.
